# Why do health workers give anti-malarials to patients with negative rapid test results? A qualitative study at rural health facilities in western Uganda

**DOI:** 10.1186/s12936-015-1020-9

**Published:** 2016-01-11

**Authors:** Robin Altaras, Anthony Nuwa, Bosco Agaba, Elizabeth Streat, James K. Tibenderana, Clare E. Strachan

**Affiliations:** Malaria Consortium, Plot 25 Upper Naguru East Road, PO Box 8045, Kampala, Uganda; National Malaria Control Programme, Ministry of Health, Kampala, Uganda

**Keywords:** Anti-malarial, Malaria, Overprescription, Provider decision-making, Patient expectations, Qualitative, RDT, Rapid diagnostic test

## Abstract

**Background:**

The large-scale introduction of malaria rapid diagnostic tests (RDTs) promises to improve management of fever patients and the rational use of valuable anti-malarials. However, evidence on the impact of RDT introduction on the overprescription of anti-malarials has been mixed. This study explored determinants of provider decision-making to prescribe anti-malarials following a negative RDT result.

**Methods:**

A qualitative study was conducted in a rural district in mid-western Uganda in 2011, ten months after RDT introduction. Prescriptions for all patients with negative RDT results were first audited from outpatient registers for a two month period at all facilities using RDTs (n = 30). Facilities were then ranked according to overall prescribing performance, defined as the proportion of patients with a negative RDT result prescribed any anti-malarial. Positive and negative deviant facilities were sampled for qualitative investigation; positive deviants (n = 5) were defined ex post facto as <0.75 % and negative deviants (n = 7) as >5 %. All prescribing clinicians were targeted for qualitative observation and in-depth interview; 55 fever cases were observed and 22 providers interviewed. Thematic analysis followed the ‘framework’ approach.

**Results:**

8344 RDT-negative patients were recorded at the 30 facilities (prescription audit); 339 (4.06 %) were prescribed an anti-malarial. Of the 55 observed patients, 38 tested negative; one of these was prescribed an anti-malarial. Treatment decision-making was influenced by providers’ clinical beliefs, capacity constraints, and perception of patient demands. Although providers generally trusted the accuracy of RDTs, anti-malarial prescription was driven by perceptions of treatment failure or undetectable malaria in patients who had already taken artemisinin-based combination therapy prior to facility arrival. Patient assessment and other diagnostic practices were minimal and providers demonstrated limited ability to identify alternative causes of fever. Provider perceptions of patient expectations sometimes appeared to influence treatment decisions.

**Conclusions:**

The study found high provider adherence to RDT results, but that providers believed in certain clinical exceptions and felt they lacked alternative options. Guidance on how the RDT works and testing following partial treatment, better methods for assisting providers in diagnostic decision-making, and a context-appropriate provider behaviour change intervention package are needed.

## Background

Malaria rapid diagnostic tests (RDT) allow countries to provide access to accurate malaria diagnosis in even the most remote areas, by means of a relatively simple to use, point-of-care test [1]. The World Health Organization (WHO) now advocates a universal ‘test, treat and track’ strategy, which recommends confirmatory parasite-based diagnosis in all patients suspected of having malaria before treating according to test results [2]. The aim is to improve quality of care, as well as to avoid overdiagnosis of cases which can lead to inappropriate or delayed treatment, reduce wastage of anti-malarial drugs, and enable effective malaria surveillance, of critical importance in the context of declining malaria mortality [2, 3]. In recent years, many national malaria control programmes across Africa have adjusted treatment policies to restrict anti-malarial prescription to patients with parasite-based diagnosis and have begun the process of scaling up diagnostic testing across the public health system [3].

The current Uganda Malaria Reduction Strategic Plan (2014–2020) recommends parasite-based diagnosis with microscopy or RDTs as part of malaria case management at all health facility levels for all age groups, and for targeted groups (children under five) at the community level [4]. RDTs are to be used to extend the access frontier at Health Centre (HC) III’s without functional microscopy, HC II’s and at the community level. Since 2005, the Uganda malaria treatment policy has specified artemisinin-based combination therapy (ACT), specifically artemether-lumefantrine (AL) (Coartem^®^), as the first-line treatment for uncomplicated malaria, with artesunate-amodiaquine (AS/AQ) as an alternative [5]. While the existing diagnostic and treatment policies have been rolled out in public health facilities across the country, adherence to policy remains suboptimal with assessment of clinical symptoms continuing to drive some malaria diagnosis and mixed reports on treatment according to test results. Like many other malaria-endemic countries, Uganda regularly faces chronic ACT stockouts [6], and malaria overdiagnosis and the overprescription of ACT have long been widely acknowledged problems [7]. As malaria prevention activities are scaled up—Uganda completed a universal coverage distribution of long-lasting insecticide-treated nets (LLINs) in 2014—appropriate clinical decision-making around diagnosis and treatment becomes even more critical given the range of causes of fever and the importance of accurate case data for progress monitoring and surveillance.

For providers working in remote areas with limited means, the introduction of RDTs has denoted a major shift from the long-standing practice of treating all fevers presumptively as malaria. If the benefits of rapid diagnostic testing are to be accrued, providers must believe in the validity of, and treat according to, test results. Parasite-based testing can be empowering, allowing providers to confidently diagnose patients with malaria. However, in many contexts, the majority of patients presenting with fever will test negative [1, 8]. How these patients are managed—what diagnosis and treatment they receive (if not malaria)—is one of the key questions associated with RDT introduction. Yet, relatively little is known about how providers construct meaning from the test results in practice and how they integrate this information into clinical decision-making processes.

To date, evidence on the impact of introducing RDTs on the overprescription of anti-malarials has been mixed, with some significant success stories, but also a broad set of challenges emerging across a number of countries. Studies in clinical settings have reported a full range of adherence to negative test results, with anywhere from 4 to 85 % of RDT-negative patients treated for malaria [9–19]. Other studies have indicated a steady adoption of ‘test and treat’ strategies as health provider reluctance and other disenabling factors are overcome [20, 21]. In Uganda, two studies have reported moderate adherence (25–35 %) to test results [22, 23], with some evidence that compliance may improve over time with increased use and experience [22].

In part, the wide range of results with regards to test adherence may reflect variations in formal influences (such as guidelines, training and support packages) across contexts and over time [24]. Some early studies observed that the lack of clear and consistent guidelines on parasite-based diagnosis may have contributed to provider tendencies to sometimes ignore RDT results [14, 25–27]. A few recent studies have attributed high prescriber compliance to quality training of clinicians and the provision of adequate support [11, 17]. However, across studies, there has been no consistent pattern indicating that length of training alone is associated with prescribing adherence; half-day and one-day trainings have preceded excellent adherence [10, 11] and a three-day training preceded extremely poor adherence [9]. In any case, the process of behaviour change from supportive interventions is neither linear nor rational, when external parameters and expectations are applied, and small differences in context, convenience, and salience have large effects on the crucial choices that health workers may make [28].

Clinical decision-making is complex, and a variety of informal influences (peers, personal experiences, perceptions and beliefs) may also impact treatment decisions, potentially undermining the impact of RDT training and support programmes. A growing body of qualitative research has begun to explore provider decision-making in conjunction with RDT use [29–33]. Interactions with peers and patients [32, 34], providers’ own learnt experience using RDTs [32], a preference for clinical diagnosis [33] and provider perceptions about the reliability of the test (combined with the recognition of malaria symptoms) [31] have been shown to influence anti-malarial prescribing decisions in various contexts. While trust of test results has been a recurrent theme, there has also been some evidence that provider capacity to confidently diagnose and treat non-malarial fevers may be influential in prescribing decision-making. A recent study from a microscopy setting found that patients who test negative do not often get an actual diagnosis and that this ‘absence of diagnosis’ may reinforce presumptive treatment practices [29].

Evidence suggests that context-specific behavioural and social factors can be influential [28] and can affect the practical success of a malaria diagnostic policy. To date, there has been little qualitative study of provider prescribing decision-making following a negative RDT result in Uganda. An early feasibility study assessing RDT acceptance and use found that providers were not confident about test accuracy, with just less than half believing that a negative RDT result excluded malaria [22, 27]; 40 % of providers reported that they would not rule out prescribing anti-malarials to patients who tested negative [27]. However, this study was conducted in 2007, prior to the full national policy change recommending parasite-based diagnosis for all age groups and some providers in the study had as little as one month experience in using the test.

A deeper understanding of factors driving prescribing behaviour in the Ugandan context is needed in order to develop optimal training and supporting interventions for successful scale-up of RDT use throughout the health system. This study aimed to address this gap with an in-depth qualitative investigation of how providers manage patients who test negative and what informs their decision to prescribe anti-malarials during routine practice in a remote, rural health care setting. The results are useful for guiding revisions to RDT training programmes and clinical and nursing school curricula, as well as for designing appropriate supporting interventions. The findings also have broader implications for clinical practice and the development of methods for diagnostic decision-making in contexts with limited means for diagnosing non-malarial fevers.

## Methods

### Study setting

This study was carried out as part of a larger project which introduced RDTs at 88 public, lower level health facilities in rural mid-western Uganda in 2011. Of the five districts of implementation, the largest district, Kibaale, was selected for study. Kibaale district borders the south-western end of Lake Albert and has an estimated population of 613,200. The district has a referral hospital and 51 public or private-not-for-profit health facilities, as well as a number of private drug shops. RDTs were introduced into 30 lower level public health facilities lacking functional microscopy for malaria. The Ministry of Health selected a histidine-rich protein 2 (HRP-2) based RDT for deployment (SD BIOLINE malaria Ag-Pf, SD 05FK60), which had passed national selection criteria and was confirmed through WHO/Foundation for Innovative New Diagnostics (FIND) panel testing. Independent field evaluation of this RDT in a country with similar endemicity showed high sensitivity (92.3 %) and specificity (82.2 %) for *Plasmodium falciparum* [35], the cause of over 95 % of infections in Uganda [36]. Prior to RDT introduction, malaria was the most common outpatient diagnosis in the district (unpublished data), accounting for more than half of all outpatient visits. RDTs were introduced during January and February 2011, starting with a training phase. Using a two-step cascade training model, district trainers were first trained in a five-day ‘training of trainers’ course before subsequently training 240 clinicians and laboratory assistants over a two-day period. Mop-up training was conducted in April 2011. The training followed the recently updated, standard government RDT training curriculum and was led by National Malaria Control Programme (NMCP) personnel (two national trainers) with support from non-governmental organisation (Malaria Consortium) staff. Following training, all health facilities received an immediate follow-up visit, followed by supervision at six weeks and thereafter on a quarterly basis.

### Sampling

Given the study’s focus on the drivers of behaviour for providers who prescribe anti-malarials to patients who test negative, health facilities were purposively sampled according to ‘prescribing performance’. Using outpatient registers, prescriptions were audited for a two month period (July–August 2011, approximately 6–7 months following RDT introduction), for all patients with a negative RDT result at the 30 health facilities. Health facilities were then ranked according to the overall proportion of patients with a negative RDT result who were prescribed an anti-malarial. Three groupings were defined according to imposed cut-off points within the data: (1) health facilities with the lowest proportion of RDT-negative cases prescribed anti-malarials were categorized as ‘positive deviants’; (2) facilities with the highest proportion were categorized as ‘negative deviants’, and (3) facilities falling in the range between the positive and negative endpoints were categorized as ‘middle performers’. Positive and negative deviant facilities were selected for qualitative investigation, with the aim of exploring the maximum range of provider prescribing behaviours.

All prescribing clinicians at the targeted health facilities were identified through interview with the health facility clinician in charge. Prescribing clinicians were defined as having consulted patients for at least ten days in the previous month. Descriptive information on provider cadre, age, and length of service at health facility was collected following the prescription audit. A total of 29 providers were identified and targeted for observation and interview. The aim was to observe three fever cases for each prescribing provider. Patients were selected for observation on a rolling basis at the point of care, in accordance with the observation procedures described below.

### Observation and provider interviews

Observations and interviews were carried out in November and December 2011, approximately ten months following RDT introduction, a cross-sectional point when their use was expected to have been integrated into routine practice. Observation and semi-structured interview guides were developed and pre-tested at health centres in two neighbouring districts. Four social scientist research assistants with a range of language skills for the context were recruited and received two days training. Two of the four research assistants conducted observation. To reduce inter-observer variability, the two observers simultaneously observed three cases and then systematically compared transcripts for discrepancies during the preparatory phase.

Data collection commenced with observation. Each observer was assigned to one provider at the health facility per day. All attempts were made to ensure that providers continued ‘normal, everyday practice’. So as not to disrupt patient flow, continuous observation of the provider was conducted until presentation of a fever case. Observers did not record notes during the first two patient consultations (whether febrile or non-febrile cases), allowing time for the provider to become habituated to the observer’s presence. Thereafter, observation notes were taken only for febrile cases. Age strata were not identified in advance to avoid disrupting the natural process of patient care; it was expected that the resulting sample would contain a sufficient range of adult and child patients. Unaccompanied minors (less than 18 years) were not observed. The observation guide covered all aspects of a typical visit for a patient with fever, including taking of patient history and physical exam, introduction to test, test procedure, communication of and reaction to test, diagnosis and treatment decision and process, and overall patient-provider interaction. Observers were instructed to record everything that was said or done, exactly as it happened, noting the time at key intervals. Observers also documented if there was anything they were unable to observe. Diagnosis, patient complaint, RDT result, and prescription information were also abstracted from the patient’s record book prior to the patient’s departure from the health facility.

Provider interviews were conducted once all observations of the provider were complete. Scope of enquiry included demographic characteristics, training received, organisation of service delivery, adjustment and changes since arrival of RDTs, process of and opinions on approaches to fever case management, supervision of and feedback received on use of RDTs and their overall experiences in using RDTs. Provider interviews were audio-recorded and conducted in English (the training language of Ugandan health workers), though there was flexibility if providers preferred to express themselves in another language.

Observation notes and verbatim interview transcripts were prepared in the field throughout data collection. To avoid loss of meaning and interpretation bias, key terms in local languages were retained alongside the English translation of observed patient-provider dialogue.

### Data analysis

Prescription data were compiled and cleaned in EpiData 3.1 and analysed using Stata 12.0. Thematic analysis of transcribed observation and interview data followed the ‘framework’ approach [37], whereby a pre-existing coding frame was developed based on the scope of enquiry to which codes were added on review of the data. All data were coded and indexed in Excel (Microsoft) and analysed according to the most salient themes.

### Ethics, consent and permissions

The Uganda National Council for Science and Technology granted ethical clearance for the study (UNCST HS 1009). For observations, informed oral consent was obtained from all providers. To avoid altering routine practice, patients and caregivers were consented for observation at the discretion of the clinicians, guided by an informed consent statement. A separate informed consent was subsequently obtained prior to provider interview. Patients and caregivers also orally consented for patient record review post observation. Consent statements included information on the broad aims of the study, confidentiality, respondent rights and uses of the data.

## Results

### Prescription audit

A total of 8344 RDT-negative patients were recorded at the 30 health facilities using RDTs for the two month period. 3151 (37.8 %) patients were identified at HCIIs and 5193 (62.2 %) at HCIIIs. Fifty-three patients had no treatment information in the outpatient register (either no prescription or prescription was not recorded). Table 1 describes the percentage of prescriptions containing anti-malarials and antibiotics. Anti-malarials were prescribed in 339 episodes of the total 8344 RDT-negative cases registered (4.1 %). Slightly less than half of these were prescribed artemether/lumefantrine (n = 151, 1.8 % of total). Four prescriptions contained more than one anti-malarial. There was no difference in the percentage of prescriptions containing anti-malarials between HCIIs (n = 178, 5.7 % of HCII cases) and HCIIIs (n = 159, 3.1 % of HCIII cases). Antibiotics were prescribed in 6509 RDT-negative patient encounters (78 %). The most commonly prescribed antibiotics were trimethoprim-sulfamethoxazole (cotrimoxazole) and amoxicillin. Of those who were prescribed an anti-malarial, 65.2 % (n = 221) were also prescribed at least one antibiotic.

**Table 1:** Results of the prescription audit (% RDT-negative prescriptions containing), spanning a two month period at 30 health facilities, 2011

	N	% of total
Total RDT-negative patients	8344	100.0 %
Anti-malarials	339	4.1 %
Artemether/lumefantrine (AL)	151	1.8 %
Sulfadoxine/pyrimethamine (SP)	141	1.7 %
Quinine (tabs or injection)	51	0.6 %
Total anti-malarials prescribed	341	n/a
Prescriptions containing more than 1 anti-malarial^a^	4	n/a
Antibiotics	6509	78.0 %
Trimethoprim-sulfamethoxazole (cotrimoxazole)	4253	51.0 %
Amoxicillin	1195	14.3 %
Metronidazole	978	11.7 %
Total antibiotics prescribed	8227	n/a
Both an anti-malarial and an antibiotic	221	2.7 %

^a^2 prescriptions contained both AL and SP; 1 AL and quinine; 1 quinine and SP

**Fig. 1:**
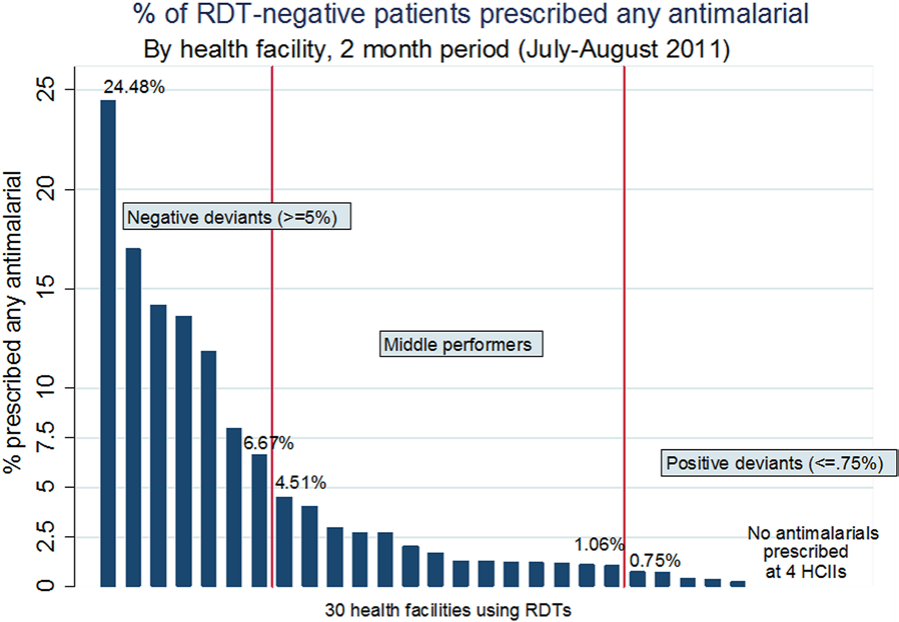
Ranking and categorisation of health facility prescribing performance

Figure 1 shows the distribution of the percentage of prescriptions containing anti-malarials by health facility. The cut-off points for health facility selection were set at less than 0.75 % of patients with negative test results prescribed any anti-malarial (‘positive deviants’) and greater than 5 % of RDT-negative patients prescribed any anti-malarial (‘negative deviants’). Given the identification of a relatively large number of ‘positive deviant’ health facilities for whom the percentage of RDT-negative prescriptions containing anti-malarials was very low or zero, a sub-set was selected for qualitative investigation, according to a range in geographical distribution (county) and health facility level. Of the 30 health facilities using RDTs, a total of 12 facilities (seven negative deviants and five positive deviants) were selected for qualitative investigation.

### Study participants

Twenty-two providers were observed at 12 public health facilities (seven HCIIs and five HCIIIs), distributed across ten sub-counties. A total of 55 fever cases were observed (22 at HCIIs and 33 at HCIIIs); the mean number of cases observed per clinician was 2.5. Three providers were observed only once due to the conduct of community outreach activities and low attendance. The characteristics of the 22 providers observed are shown in Table 2. Most providers were of lower cadres (nurses or nursing assistants) and slightly more were female than male. The median age of observed providers was 37 years (age range of 26–59 years). All but one of the providers had participated in formal training on the use of RDTs (the remaining provider, a nursing assistant, received on the job training from the facility in-charge.) Most providers also reported having participated in at least one other training or seminar which included a patient care focus (mostly case management trainings for a range of infections) within the past 12 months.

**Table 2:** Characteristics of providers observed in the study

	Number of providers (%)		
	HCII	HCIII	Total
Gender			
Female	6	8	14 (64 %)
Male	4	4	8 (36 %)
Cadre			
Nursing assistant	5	4	9 (41 %)
Nurse	4	7	11 (50 %)
Clinical officer	1	1	2 (9 %)
Year of last qualification			
1991 and before (more than 20 years prior)	3	0	3 (14 %)
1992–2001 (10–19 years prior)	0	3	3 (14 %)
2002–2011 (within last 10 years)	7	9	16 (73 %)
Number of years at health facility			
Less than 1 year	2	1	3 (14 %)
1–5 years	5	8	13 (59 %)
6 or more years	3	3	6 (27 %)
Age			
30 and younger	4	5	9 (41 %)
31–45	1	7	8 (36 %)
46 and older	4	0	4 (18 %)
Total	10	12	22

All of the 55 observed patients were tested for malaria with an RDT. Slightly more than two-thirds (n = 38) tested negative (Table 3). Negative results were not evenly distributed across age groups; 71 % of all observed RDT-negative patients were adults. The majority of observed patients were female (35, 63.6 %). At least one RDT-negative case was observed at each health facility; however, 40 % of the total RDT-negative case observations occurred at two HCIIIs. 53 % of all observed cases were managed by nurses, 40 % by nursing assistants and 7 % by clinical officers. The average visit length (time from beginning of consult to treatment dispensing) was 1 h 17 min (range of 10 min to 3 h 35 min).

**Table 3:** RDT results of observed patients by age group

	RDT-negative	RDT-positive	Total
	N (%)	N (%)	N (%)
Adult	27	6	33 (60.0 %)
Child	11	11	22 (40.0 %)
Total	38 (69.1 %)	17 (30.9 %)	55 (100.0 %)

### Observed prescription of anti-malarials

Provider adherence to test results was high across all observed patients. Of the 38 observed patients with a negative RDT result, one was prescribed an anti-malarial (quinine). No observed RDT-negative patients were prescribed AL. All observed RDT-positive patients were prescribed anti-malarials.

### Themes influencing decision-making

Analysis of observation data and provider interview transcripts identified a number of factors that appeared to affect provider decision-making to prescribe anti-malarials to patients who tested negative. These were grouped into three intersecting thematic areas: clinical beliefs (what providers believe is the right thing to do), capacity constraints and the ability to make an alternative diagnosis (what providers have the means to do), and perception of patient demand (what providers think the patient wants them to do). Each of these themes is described in detail in the sections that follow.

### Clinical beliefs: trusting results

Providers generally appreciated the importance of parasite-based diagnosis; almost all providers acknowledged that it is not easy to accurately diagnose malaria based only on symptoms and signs. Drawing on their own experience and training, most providers reported that they trusted the accuracy of the test, noting, for example, that even patients who tested negative generally improved. Most providers appeared to accept that a negative RDT result means the patient does not have malaria and that fever may be symptomatic of an alternative illness or infection, though this was not universal. There was high awareness of the national parasite-based diagnostic policy and the requirement to restrict anti-malarials to patients who test positive.

“I cannot give out [AL] unless the RDTs have turned positive.” [HW02, Nurse-midwife, HCIII]

A few providers did however raise doubts over test accuracy, citing patients who tested negative by RDT and then later positive by blood smear examination, which they sometimes attributed to “*other strains of malaria*” that could not be detected by the RDT. These may have been examples of the ‘prozone effect’ [38], whereby the RDT can give a negative result due to an excess of antigens or antibodies, a phenomenon which appears to be specific to HRP-2 tests. Two providers also mentioned doubts due to the time elapsed prior to reading test results.

“Generally I also have some doubts because one time my child fell sick, had all symptoms of malaria, when I did an RDT test, it turned out to be negative. Then the next day, I repeated the test, it again showed negative. Then I took the baby to hospital and that very night, the baby convulsed, they tested her… and the baby had malaria plus plus. Automatically, we put the baby on quinine IV, and the baby improved. So because of that experience, I have some doubts about the RDT results because that was really scaring and worrying.” [HW02, Nurse-midwife, HCIII]

### Clinical beliefs: “you cannot get the parasites”

**Fig. 2:**
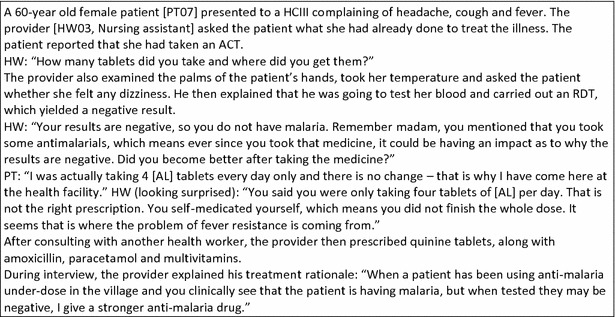
Observation notes: RDT-negative patient prescribed an anti-malarial

In line with observed practice, providers by and large reported that they prescribed anti-malarials to RDT-negative patients in rare circumstances, if at all. When they did so, it was driven by a clinical rationale. For example, in cases where patients had already taken ACT prior to coming to the health facility, some providers seemed to presume treatment failure due to resistance, even when there was no prior confirmation that the patient had malaria. In such cases, providers reported shifting ‘automatically’ to prescribing a second-line anti-malarial (which at the time of study in Uganda had officially changed to dihydroartemisinin piperaquine for uncomplicated malaria, with quinine tablets remaining the alternative and more readily available second-line treatment). The one observed patient who was prescribed an anti-malarial despite a negative test result, was prescribed quinine, after having reported taking a partial dose of AL prior to coming to the health facility (Fig. 2).

Providers appeared to reconcile the need for anti-malarial treatment with the negative RDT result, noting that in these cases (after taking ACT) “*you cannot get the parasites”*. The specificity of provider rationales seemed to suggest a limited understanding of how the antigen-based test works (what exactly the test identifies), rather than more pervasive doubts about test accuracy. In cases where providers reported presumptively treating malaria, providers did not mention the diagnostic possibility of a non-malaria febrile illness. However, there was no evidence to suggest that the prescription of anti-malarials precluded the prescription of other treatments.

“There are some times when I give quinine injection when the patient has signs like shivering, vomiting, headache, very high temperature, etc. yet the patient had taken [AL], but is still very sick. At that point I can give quinine injection even if the RDT was negative because in most cases if the patient takes any anti-malaria drugs before testing, there is a possibility of the results turning out negative. However, that does not mean that the patient doesn’t have malaria. So, in such instances, I give second line treatment which is quinine… In most cases it’s the patient history and examination that gives an option to give anti-malarial drugs or not.” [HW20, Nurse in charge, HCII]

### Capacity: “treat what you know”

Providers generally felt that the use of RDTs was a positive, enabling change that enhanced their capacity to provide quality care; specifically, they no longer had to rely on “*guesswork*” and could now “*treat what you know*”.

“At least one treats what one is knowledgeable about and not depending on guesswork.” [HW12, Nursing Assistant, HCIII]

A couple of providers explicitly acknowledged improvements in patient outcomes, noting that when they treated presumptively, patients failed to improve because they were not treating the right illness. One tied this to making it easier to identify treatment failures and knowing when to prescribe a second-line treatment. A number also mentioned that RDT use helped to reduce drug resistance and ACT wastage. Some providers also reported a sense of pride from practicing new skills and increased confidence in managing patients.

“The main importance is to really know what we are treating… for all the years we have been treating people, many complain of fevers and yet they are suffering from other illnesses. Secondly, RDT testing has helped us a lot in controlling the community. Those days they would ask for antimalarial drugs by saying ‘“Musawo” [health worker, in Runyoro-Rutooro], give me some drugs for my children at home, they are suffering from fever’ but [now], be it here or any bigger health facility, they first have to carry out test before giving you treatment”. [HW09, Nursing Assistant, HCII]

### Capacity: limited patient assessment and diagnostic practices

While providers appeared to value RDTs as a confirmatory diagnostic, in observed practice they demonstrated limited capacity to diagnose non-malarial fevers. Providers collected minimal information from the patient, rarely taking a patient history (beyond key complaint or symptoms and their duration), conducting physical examinations, or enquiring about the presence of specific symptoms which could be associated with common causes of fever. In a number of cases there was simply no consultation beyond the initial registration of the patient (name, age, village and patient complaint were requested and recorded in the patient’s record book). Following a negative RDT result, providers were observed to take some additional diagnostic action in about a quarter of cases (distributed across providers). These actions were: asking additional questions, generally reflecting a lack of initial history taking (e.g. *“Does he have cough?”);* offering advice or suggesting the need for additional testing, but without referral (e.g. *“If you think you have typhoid you have to go and seek treatment from elsewhere”)*; and referring to a higher level health facility for additional testing and care (one child presenting with danger signs, two adults for typhoid testing).

Providers did not formulate clear diagnostic statements, with recorded information being generally limited to patient complaints, RDT result and prescribed drugs. Providers were observed to be performing largely as drug dispensers. The common response to a negative RDT result was to simply prescribe for other symptoms present (e.g. cough), with the prescriber typically treating the test result and each patient complaint independently. Although patients were not systemically sampled for observation (and RDT-negative and RDT-positive patients were not comparable with regards to age distribution), on average, slightly fewer drugs were prescribed for the observed RDT-negative patients: 2.94 drugs per prescription versus 3.41 for RDT-positive patients. Overall, antibiotic prescription was high across both positive and negative patients: 45 of the 55 observed patients were prescribed at least one broad-spectrum antibiotic. Just one of the 38 patients who tested negative was prescribed only an antipyretic.

### Capacity: constraints

Providers’ limited ability to make an alternative diagnosis for patients with negative RDT results appeared to be associated with three main constraints: a lack of know-how and low level of clinical skills, a lack of other point-of-care diagnostics and insufficient time due to understaffing and high workloads.

Providers generally knew what they should do following a negative result; most reported that they review the patient history or investigate other causes of fever. Most also mentioned referral or requesting the patient return if no improvement as possible courses of action. However, knowledge of what to do following a negative result commonly appeared more theoretical than practical (providers had ‘knowledge that’ they should investigate further, but had less ‘knowledge how’ to go about doing so). Some vaguely mentioned that they try to “*find out other causes of fever*”, without describing with any specificity how they go about doing so. A few providers only mentioned that they “*treat other complaints*” and a small group (primarily nursing assistants) focused on patient communication, noting that they reassure the patient or explain that other things can cause fever. Underscoring the observed lack of diagnostic statements, many providers expressed simplistic ideas about febrile illness. Most providers tended to conflate symptoms and disease aetiology; cough and headache were frequently referred to as “*causes of fever*”. A few providers did not accurately express illness concepts, misusing terms such as fever, malaria and high body temperature. This practice was likely an artefact of illnesses classifications in local languages—although malaria is more precisely translated into Runyoro-Rutooro (a dominant language of the region) as “*omuswijja gw’emibu”*, a single word, “*omuswijja*”, is frequently used to mean both fever and malaria—but also seemed indicative of possible clinical confusion, particularly amongst older nursing assistants (40, 52 years).

“[A negative result] means that the patient does not have any fever, but could be having other infections like wounds or cough… When the results are positive, I tell the patient, I have tested you and found out that you have got fever (“omuswijja”)… In case a patient is having negative RDT results, I tell them that ‘we have tested your blood and you do not have fever (“omuswijja”), so which means it is either the cough or headache causing the fever depending on the patient’s other complaints. If it is cough, I then give them medication for cough and assure them that they will be fine”. [HW15, Nurse in charge, HCII, 52 years]

Providers also reported limited to no use of clinical guidelines to aid in diagnostic decision-making, an attestation which was validated in observed practice. In training, providers were advised to refer to the ‘Integrated Management of Childhood Illness’, ‘Uganda Clinical Guidelines’ and a list of treatment protocols for common causes of fever provided in the national ‘RDT User’s Manual’. Although most providers reported that they had at least one of these guidelines readily available, most also forthrightly explained that they did not refer to them in practice. One explained (quite accurately) that he did not have any specific guidelines to aid in diagnosing non-malarial fevers or managing patients who tested negative. The lack of other diagnostic tools or ability to test for other diseases (typhoid in particular) was also cited as a constraint by providers.

Most providers complained about the high workload associated with using RDTs. Providers reported that they were now doing additional tasks and that it required “*too much time per patient to manage properly”*, particularly in a context where they were often working alone to manage an “*overwhelming number of patients”*. However, some providers noted how this additional time spent with the patient was beneficial: “*If somebody comes with different complaints, at least now I interact with them at length, I talk with patients”. [HW02, Nurse*-*Midwife, HCIII].*

In spite of these reported challenges, a belief in doing the right thing for the patient (responsibility to treat) and a desire to ration ACT use appeared to override the workload constraint.

“Much as the work is too much for me, I think it’s better than treating what I don’t know.” [HW05, Nursing Assistant, HCIII]

### Capacity: “nothing else we can do”

In the absence of an alternative diagnosis, a small number of providers reported that they sometimes prescribed anti-malarials in part because they perceived no other options to be available. Although most providers explained that they referred the patient or first consulted with others at the health facility when they were unable to determine cause of fever, two mentioned sometimes treating RDT-negative patients with an anti-malarial when they were unable to make an alternative diagnosis. These providers’ perceived lack of options appeared to result from a lack of confidence in an alternative diagnosis/treatment and an underlying fear of missing the true diagnosis or risking death, particularly when faced with a very ill patient. This did not appear to be linked to the availability of other treatments. In addition, some providers also suggested that “*other health workers”* (particularly those with lower qualifications) prescribed anti-malarials to patients who test negative because they lack the confidence or skills to manage the patient properly.

“At times we treat because you see all the signs of malaria are there, so in such cases we treat because there is nothing else we can do… Also when you have volunteers and they lack experience and when patients come they just give out the [AL] because they cannot further to establish the possible cause of fever”. [HW11, Nurse, HCIII]

“There are times when I am forced to give them antimalarials, especially when the sickness is too much and they become better later… even when the RDT results are negative… But if the situation persists, I then refer.” [HW18, Nursing Assistant, HCII]

“…my colleagues give it [AL] out due to lack of confidence and when the patient is looking so sickly they just treat. In the recent past we have met and agreed that if you are not confident, refer to microscopy or just be confident that the drug you have prescribed will heal the patient… I cannot deny the truth, my colleagues here do it and the reasons could be lack of confidence and they think they have missed proper diagnosis”. [HW01, Nurse in charge, HCIII]

### Perceptions of patient demand

Providers commonly viewed “*patient refusal”* as a major challenge and often perceived refusal as relating to the test and a desire for AL. Most providers reported that they had been pressured to prescribe anti-malarials to RDT-negative patients at some time and that patient reactions to RDTs affected their work (e.g. more time to counsel and explain results to patients).

“The most challenging part is communicating the results to the patient because some patients come here with a belief that they have malaria and expect to get treatment, so telling them a negative result is a disappointment on their side”. [HW20, Nurse in charge, HCII]

Although providers almost universally complained that patient reactions were a challenge, most were adamant that they did not allow this to influence their prescribing practices. Nonetheless, perceived patient acceptance was indirectly cited by a few providers as a reason for giving anti-malarials to patients who tested negative: Two providers (both nursing assistants) reported sometimes giving SP to placate RDT-negative patients or because they believed a patient should not go away empty-handed. This acquiescence to perceived patient demand appeared to be conflated with a ‘desire to treat’ and help the patient (providers wanted to provide treatment, but may have lacked the capacity to identify the right treatment for the patient). Others mentioned “*patient pressure*” as a factor influencing their colleagues’ behaviour.

“The most challenging thing is patients rejecting results and it’s in those kinds of instances that I decide to give [SP]”. [HW19, Nursing Assistant, Acting in charge, HCII]

“There was one time when a man came and he wanted to be given [AL]. When he arrived he never even went for diagnosis but moved direct to the dispensing window and started demanding for [AL]. We all refused and convinced to have him tested first and, upon testing, he was negative. After refusing to give him the [AL] he came back the following day complaining and we had just to give him and he did not come back again”. [HW10, Nurse in charge, HCIII]

## Discussion

While there was some range in prescribing performance observed across health facilities, the study found much higher overall adherence to test results than observed in earlier studies in Uganda [22, 23]. This could in large part be due to changes in national policy recommendations – earlier studies were conducted prior to the full national policy change recommending parasite-based diagnosis for all age groups—and in the composition of training and support packages. In this setting of routine use, providers underwent specific training on the clinical management of patients who test negative and received regular supervision, with clear guidance that they should not prescribe anti-malarials to patients who tested negative; this likely curtailed misuse. The timing of data collection (conducted once the programme was established, rather than immediately following or coinciding with RDT introduction) may also have affected findings. Earlier studies assessed provider views and experiences shortly after implementation, which may not have allowed sufficient time for providers to form opinions on the role of RDTs in diagnostic and treatment decision-making [27]. While it has been suggested that provider adherence improves over time [20–22], it has also been observed that initial improvements may not be sustained over the longer-term [13]. However, it would seem likely that these effects are mitigated by the ongoing presence or absence of supporting interventions. A desire to ration ACT in a context of recurrent stockouts, combined with high awareness of the national treatment guidelines restricting ACT prescription to patients who test positive, may also have limited prescription of anti-malarials to patients who tested negative. However, it is also possible that providers under-recorded anti-malarial prescription to patients who tested negative, knowing that this deviated from guidelines and that outpatient records may be reviewed by supervisors.

While adherence to test results was high, this study found some key interplaying factors influencing providers to prescribe anti-malarials to patients who tested negative. Some anti-malarial prescription was driven by perceptions of treatment failure or undetectable malaria in patients who had already taken ACT prior to coming to the health facility. Capacity constraints, including a lack of provider know-how, time, and alternative diagnostics, appeared to considerably affect provider ability to make an alternative diagnosis. Limited routine diagnostic practices (i.e. history taking, physical examination) meant that providers collected little information that would aid them in treatment decision-making. Perceptions of patient demand for treatment, coupled with perceived patient rejection of test results, also appeared to result in some anti-malarial (SP) prescription to patients who tested negative.

The particular issues which arose relating to testing and treating following ACT use require further consideration. Some providers reported ‘automatically’ shifting to a second-line treatment (quinine) despite a negative test result (apparently attributing suspected treatment failure to drug resistance). In these circumstances, it appeared that anti-malarial prescription decision-making was driven by a combination of intuitive and rationalistic approaches. On the one hand, after years of presumptive treatment, it seems likely that providers remain predisposed to seeing malaria as the most likely explanation for persistent fever. Findings suggest that underlying fears of missing a diagnosis and concerns for patient safety remain influential, particularly in a remote context where the consequences of delayed treatment can be fatal. Presented with a patient with persistent fever who is manifestly ill and has already taken ACT, providers may also tend toward presumptive treatment “*just in case it may help the patient*”, as one provider put it.

At the same time, some providers applied a rationalistic approach, describing an analytic framework in which “*you cannot get the parasites*” after ACT administration. However, it was less clear what the underlying logic was for this belief. In contrast to earlier research in Uganda [22] and elsewhere [31], providers in this study did not appear to be strongly influenced by underlying doubts about the accuracy of test results. It is possible that some providers may have thought that patients who already took ACT had undetectable malaria due to low parasitaemia (since the administered ACT would have reduced the parasite load), however this was not articulated. It was also not clear how providers may have reconciled the suspected presence of low levels of undetectable parasites with the clinical presentation of a manifestly ill patient, which was frequently mentioned as the initial, driving rationale for suspecting malaria and prescribing anti-malarial treatment. Alternatively, it is possible that providers did not have a clear understanding of what the test detects. There may have been issues related to the deployment of HRP-2-based tests that influenced providers’ understanding of how the RDT works and the ability of the RDT to detect malaria following ACT use. Incidentally, there also appeared to be at least one example of a false negative RDT result due to the ‘prozone’ effect [38], concerning from the perspective of incorrect diagnoses as well as for the potential for raising distrust in the test itself.

The challenges related to an inability to diagnose and treat non-malarial fevers have been well-documented [27]. It was found that providers’ treatment decision-making for patients who tested negative was in part influenced by various capacity constraints, starting with providers’ limited clinical know-how and diagnostic skills. On occasion, some providers reported prescribing ACT as a result of capacity constraints (simply because they lacked other options). The confluence of multiple pressures—the responsibility to save lives, a high workload and low level clinical skills—appeared to foster a general ‘expediency’ approach in which providers may choose a ‘path of least resistance’. Time pressures may force providers to expedite the care process, limiting their interaction with the patient and inhibiting their ability to probe just when they need to ‘dig deeper’ for other causes of fever. An absence of diagnosis and clear alternative treatment plan, in turn, may render providers more susceptible to perceived patient pressure. These combined circumstances may influence a provider to decide that it is easier ‘to just give’ an anti-malarial.

As providers lacked specific guidelines or algorithms to aid in the diagnosis and treatment of patients who tested negative, it is also unsurprising that providers appeared to rely largely on intuitive decision-making. Given that point-of-care diagnostics for other common causes of fever to be deployed at low level health facilities are not yet forthcoming [39], there is an urgent need to develop better methods for assisting providers in diagnostic decision-making. However, despite these constraints, providers did not seem to make the most of the limited means they had available. Whether due to limited time, excessive workload, lack of confidence or ability, providers rarely appeared to make the most of their interaction with the patient to collect information that would aid them in making a differential diagnosis.

Other studies have observed a potential trade-off between the overprescription of anti-malarials and the overprescription of antibiotics in settings of RDT use [19, 40–42]. In this study, the potential overuse of antibiotics appeared to be more an indication of symptom-based, multiple prescribing practices (polypharmacy) than a trade-off associated with RDT use, although this practice may be exacerbated when the diagnosis is uncertain. Moreover, the very small number of patients observed to receive no treatment or only supportive treatment (antipyretic) suggests that providers either rarely considered self-limiting, viral infections or that they were being responsive to perceived patient expectations for drugs.

Pressure to conform to perceived patient preferences has been found to be influential in some settings [43], although some evidence has suggested that this is not a primary driver of prescription practices [44]. Providers in this study reported that patient demand for anti-malarials did not affect their prescription practices following testing, though a desire to treat the patient and a lack of other options sometimes influenced them to prescribe SP as a ‘consolation’ treatment. Replacement of ACT with SP prescription in the management of RDT-negative patients has been observed elsewhere [45] and the study’s findings are in line with earlier results from Uganda, which observed that some RDT-negative patients were given non-ACT malaria treatments, as providers felt they could thus ‘save ACT’ for those who tested positive and still meet patient expectations [27]. In this study, the decision to prescribe SP to patients who tested negative was predominantly linked to a desire to provide some treatment to the patient (so that he would not go “*empty*-*handed*”), rather than a strong belief in its efficacy or appropriateness as treatment. Providers were also very much aware of guidelines, and perhaps perceived this as a way of circumventing the policy regarding ACT use.

Table 4 summarizes the main, intersecting themes influencing providers to prescribe anti-malarials to patients who tested negative in this context, positive influencers in promoting adherence to RDT results, and potential implications for provider behaviour change, specifically the design of training and appropriate supporting interventions. Behaviour change is affected by a complex set of factors and it will be important for all interventions to be appropriately designed in consultation with targeted stakeholders to ensure relevance to their social realities.

**Table 4:** Summary of findings and potential implications for provider behaviour change

	Factors influencing anti-malarial prescription to patients who test negative	Positive influencers (may support adherence to test result)	Potential opportunities for influencing provider behaviour change
Clinical beliefs	Perceived treatment failure or undetectable malaria in patients who already took ACT (may be driven by limited understanding of how RDT works) If provider perceives ‘resistance’ or undetectable malaria, may fail to consider non-malaria febrile illness (i.e. miss true diagnosis)	Appreciation of importance of parasite-based diagnosis Trust in RDT accuracy and belief that a “*negative RDT means the patient does not have malaria*” Motivated by desire to do the right thing for the patient Motivated by reducing ACT wastage	Provide practical guidance on management of patients who have taken a partial dose of ACT (clear guidelines; may benefit from on the job training and review of context-specific cases) Clarify provider understanding of how RDT works; sharpen communication on limitations of HRP-2-based tests (including ‘prozone effect’) Improve referral guidance and appreciation of benefits (reducing unnecessary treatment, detection of treatment failures, identification of other causes of fever) Provide guidance on risk mitigation for severely ill patients, including role of RDTs
Capacity constraints (ability to make alternative diagnosis)	Limited diagnostic practices (history taking, examination); providers don’t give themselves means to identify alternative cause of fever Lack know-how, diagnostic tools, time	Satisfied to be *“treating what [I] know*”, despite workload Perceive RDT use as enabling; no longer rely on “*guesswork*” Know what to do when they get a negative result Acknowledgement of the challenge of making an alternative diagnosis	Increase awareness of the impact of missed diagnoses and overtreatment on patient outcomes (may encourage providers to invest more in the diagnostic process, maximize existing opportunities for information gathering to aid in diagnosis, and resist the urge to provide anti-malarials “*just in case*”) Develop better methods for assisting providers in diagnostic decision-making: make a negative result ‘actionable’ (i.e. clear algorithms in poster format on managing non-malaria fever cases)
Perceptions of patient demand	Perceive patient “*refusal*” as relating to test and desire for ACT		Improve interpersonal communication skills and quality of interaction with patient (may facilitate correct diagnosis and treatment) through training, support supervision or patient consultation guides/checklists

### Limitations

This study had some potential limitations. It is possible that providers may have under-recorded anti-malarial prescription to patients who tested negative, but there was no alternative data source available for validation. Similarly, providers may have under-reported what they knew to be unacceptable behaviours (including prescribing anti-malarials to patients who tested negative) during interview. Probing techniques and multiple lines of questioning were used to elicit providers’ true beliefs and behaviours and encourage providers to feel that all responses were acceptable. It is also possible that providers may have altered their behaviour (attempted to perform better) during observation because they were aware they were being watched (observer-expectancy effect). Data collection procedures, observer techniques (habituation) and training aimed to limit this effect and capture providers as they usually perform. The use of multiple methods of data collection and triangulation of data also helped to minimize any researcher bias in the overall analysis. This study largely observed providers of lower cadres (nurses and nursing assistants), which limited the possibility of observing potential differences in behaviour by provider cadre. The sample of observed cases was also relatively small, with only one RDT-negative case being prescribed an anti-malarial. Finally, it happened that there was an uneven distribution of positive and negative test results across child and adult patients, limiting opportunity to compare care practices between RDT-positive and RDT-negative patients and to interpret any potential differences between adult and child patients in terms of diagnostic or prescribing behaviour.

## Conclusions

This study found overall high provider adherence to test results, but that providers believed in certain clinical exceptions and felt they lacked alternative options following a negative result. Although providers generally trusted the accuracy of RDTs, anti-malarial prescription was driven by perceptions of treatment failure or undetectable malaria in patients who had already taken ACT prior to coming to the health facility. Capacity constraints, specifically a lack of alternative diagnostic tools, provider know-how and time, and limited routine diagnostic practices all hampered the ability of providers to identify alternative diagnoses. Perceptions of patient demand for treatment, coupled with perceived patient rejection of test results, also appeared to drive some anti-malarial prescription following negative test results. The observation that providers may replace ACT prescription with an alternative anti-malarial is important and warrants focus. Guidance on how the antigen-based tests work and testing following partial treatment doses, as well as better methods for assisting providers in diagnostic decision-making, are needed. Guidance provided should be as practical as possible to avoid leaving ‘grey zones’ which could lead to misinterpretations in clinical decision-making. Further exploration is also needed on how best to equip providers to make the most of their interaction with the patient. This has implications for organisation of care and should include exploration of how to most effectively balance the drive for efficiency with quality patient communication. A number of opportunities for influencing provider behaviour change to further adhere to negative RDT results have been suggested. Behaviour change is complex and requires a multitude of complimentary interventions relevant to the social realities of the providers, which could include review and clarification of guidelines, training, supervision, performance improvement and capacity building.

## Abbreviations

ACT: artemisinin-based combination therapy
AL: artemether/lumefantrine (*NB* providers and patients routinely referred to AL by brand name. References to the brand name were replaced with AL)
HCII: Health centre II
HCIII: Health centre III
HRP-2: histidine rich protein 2
MOH: Ministry of Health
RDT: rapid diagnostic test
SP: sulfadoxine-pyrimethamine (*NB* providers and patients routinely referred to SP by brand name. References to the brand name were replaced with SP)
